# Controlling morpho-electrophysiological variability of neurons with detailed biophysical models

**DOI:** 10.1016/j.isci.2023.108222

**Published:** 2023-10-16

**Authors:** Alexis Arnaudon, Maria Reva, Mickael Zbili, Henry Markram, Werner Van Geit, Lida Kanari

**Affiliations:** 1 Blue Brain Project, École Polytechnique Fédérale de Lausanne (EPFL), Campus Biotech, Geneva, Switzerland

**Keywords:** Health technology, Materials science

## Abstract

Variability, which is known to be a universal feature among biological units such as neuronal cells, holds significant importance, as, for example, it enables a robust encoding of a high volume of information in neuronal circuits and prevents hypersynchronizations. While most computational studies on electrophysiological variability in neuronal circuits were done with single-compartment neuron models, we instead focus on the variability of detailed biophysical models of neuron multi-compartmental morphologies. We leverage a Markov chain Monte Carlo method to generate populations of electrical models reproducing the variability of experimental recordings while being compatible with a set of morphologies to faithfully represent specifi morpho-electrical type. We demonstrate our approach on layer 5 pyramidal cells and study the morpho-electrical variability and in particular, find that morphological variability alone is insufficient to reproduce electrical variability. Overall, this approach provides a strong statistical basis to create detailed models of neurons with controlled variability.

## Highlights

• Sampling of single-cell models of neurons reproduces experimental variability
• Many neuronal models enable statistical analysis of electrophysiological properties
• MCMC sampling method allows for efficient design and sampling of electrical models

## Graphical abstract

**Figure:**
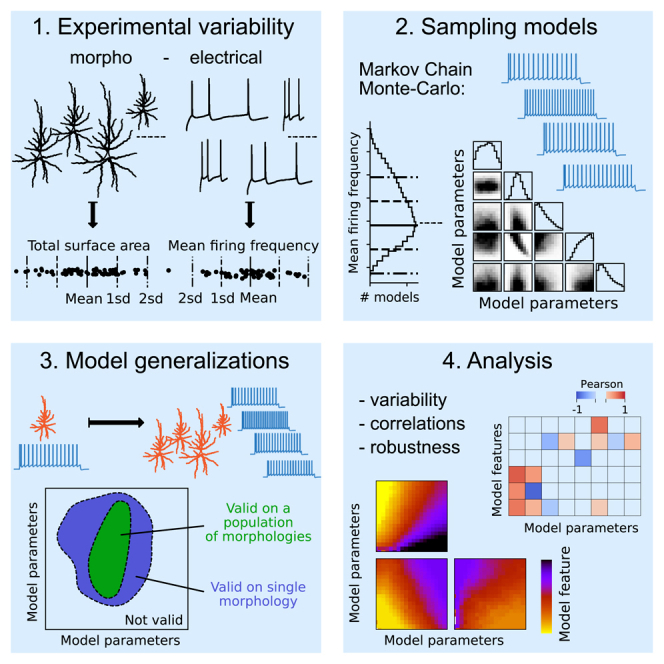

## Introduction

Neurons in the brain are highly heterogeneous, both in terms of morphologies and electrical phenotypes. Attempts have been made to classify them into morphological, electrical, or combined morpho-electrical types,^1^^,^^2^^,^^3^ however, even within a single cell type, cells are highly variable. For example, morphologies grow in the available space following the local signaling processes, hence their shapes and sizes are location-specific and unique^4^ and have an impact on electrical properties.^5^ Morphological variability has been studied in the context of robustness of structural connectivity for example by^6^^,^^7^^,^^8^ and shown to be of critical importance. Electrophysiological variability, even within a firing type is also of paramount importance^9^^,^^10^ in many aspects of brain modeling. In general, biological noise and variability have implications in a wide range of brain mechanisms such as behaviors,^11^ computation,^12^ neural responses,^13^ information processing,^14^ or for brain dynamics.^15^ Here, we focus on modeling the variability of cell firing properties for a given firing type and not on modeling other types of randomness, such as ion channel stochasticity, or synaptic noise. Firing variability has been shown to be important for a range of biological mechanisms of the brain, such as for network properties^16^, resilience to changes in synchrony with epilepsy,^17^^,^^18^^,^^19^ increased information content for efficient population coding^20^^,^^21^^,^^22^^,^^23^^,^^24^ or energy efficiency.^25^

The intra-type variability of neuronal electrophysiological properties has a biological basis but ultimately originates from the choice of the scientist to classify the recorded cells into a specific firing type. Indeed, cells often form a “continuum of types,” as visible from the often blurry limits where some cells cannot be consistently classified.^3^^,^^26^ A cell type definition should not only account for specific values of certain electrophysiological features but also for their variability. Within a cell type, this variability will differ across species, age, or even brain regions and must therefore be factored in. Hence, for modeling studies ranging from detailed single cells to circuit simulations, such a complete characterization of cell types will play an important role. First, for single-cell modeling, the unknown constraints of the feature variability on ion channel conductances can be statistically quantified by considering an ensemble of models.^9^ This is important to ensure most biologically plausible models are considered, in light of the recent interest in the inherent degeneracy of electrical models.^27^^,^^28^^,^^29^ Second, one can study the interplay between these conductances and the resulting firing types to gain a theoretical understanding of the necessary interactions between ion channels to produce a specific firing type. Additionally, it could lead to the proposition of hypotheses that could be tested experimentally. For example, these hypotheses might involve conducting “knock-out” experiments on specific channels to verify the accuracy of the proposed models, such as for example done in the Hippocampus by Roy et al.^30^ Alternatively, one can verify if the model replicates established experiments to delve deeper into their underlying mechanisms, using, for example, a gradual reduction of the target conductances. Third, having a large population of valid, but generic models, allows the selection of sub-populations with specific properties. Constraints can be imposed directly by restricting certain feature values or indirectly via certain properties of larger circuit models, or through generalization to other morphologies. Such an approach is critical to understanding the role of specific model features in a wider context, often hardly available via direct model building for complex circuit simulations. A recent attempt at combining electrical models and circuit dynamics with deep learning by Goncalves et al.^31^ was limited to simple and well-understood circuit dynamics with a few features, but not yet scalable toward more detailed circuit models.^32^^,^^33^^,^^34^^,^^35^

In this study, we sample a set of models that accurately reproduce the desired firing type on a population of morphologies taking into account its variability. This facilitates the statistical analysis of interdependencies between morphological and electrical properties as well as helps address the question of which morphological features affect specific electrophysiological properties. Finally, and most importantly, this approach allows the generation of a population of cell models with a controlled morpho-electrical variability. Several attempts to quantify the variability in neuronal parameter space have been made in the past. It began as early as,^36^ then it became central in several works more than a decade later. First, in a study by Prinz et al.^37^ they build models of lateral pyloric neurons with this sampling approach to bypass the common hand-tuning of parameters or later in a study by Taylor et al.^38^ to perform a more in-depth analysis of ion channels interactions in biophysical neuronal models. In these works, they randomly sample the parameter space and subsequently filter out models with features out of their prescribed range. Other approaches leveraging optimization algorithms^39^ for Purkinje cells or for hippocampal neurons^30^^,^^40^ to understand the shape of the neuron parameter space were attempted.

Here, we will instead use the Markov chain Monte Carlo (MCMC) method (see for example Gilks et al.^41^) to sample the parameter space of the electrical model of rat cortical layer 5 pyramidal cells (L5PC).^42^ This sampling method, already used in a similar context, but on simpler models by Wang et al.^43^ provides a tractable Bayesian framework for sampling parameters of the model, not based on machine learning algorithms, such as successfully developed by Goncalves et al. and Oesterle et al.^31^^,^^44^ It also improves on random sampling by preventing evaluations of too many models with wrong firing properties and provides statistical guarantees that the parameter space is well sampled with respect to a given probability distribution, implemented here from the cost function constructed from electrophysiological features. From these sampled models evaluated on a single reconstructed morphology, we develop a method to generalize them to a population of morphologies inspired from Hay et al.^45^ by adjusting surface areas of the axon initial segment (AIS) and soma based on relative input resistances between them and the dendrites. With these sampled and adapted models, we studied the morpho-electrical variability of the layer pyramidal cells, and specifically their ability to reproduce experimental variability.

## Results

### Experimental morpho-electrical variability

We considered two datasets: 64 morphologically detailed reconstructions of L5PC^32^^,^^46^ and 44 electrophysiological patch clamp recordings of the same cell type.^32^^,^^42^ These datasets are separate, hence the recordings cannot be matched to specific morphologies. The L5PC morphologies are classified into four subtypes according to the properties of the apical dendrite: thick-tufted, bitufted, small-tufted, and un-tufted (see STAR methods and Kanari et al.^47^). We will refer to thick-tufted the subtype denoted as TPC:A in Kanari et al.^47^ We rediametrized all the morphologies according to the algorithm presented in STAR methods to ensure that they have consistent diameter profiles. The algorithms first fit a model of the diameter as a function of distance to the furthest terminal points from the morphological population, and then assign diameters according to it. As our goal was to obtain consistent diameter profiles, this algorithm yielded satisfactory results, but more biologically based diametrization algorithms could be used.^48^

**Figure 1:**
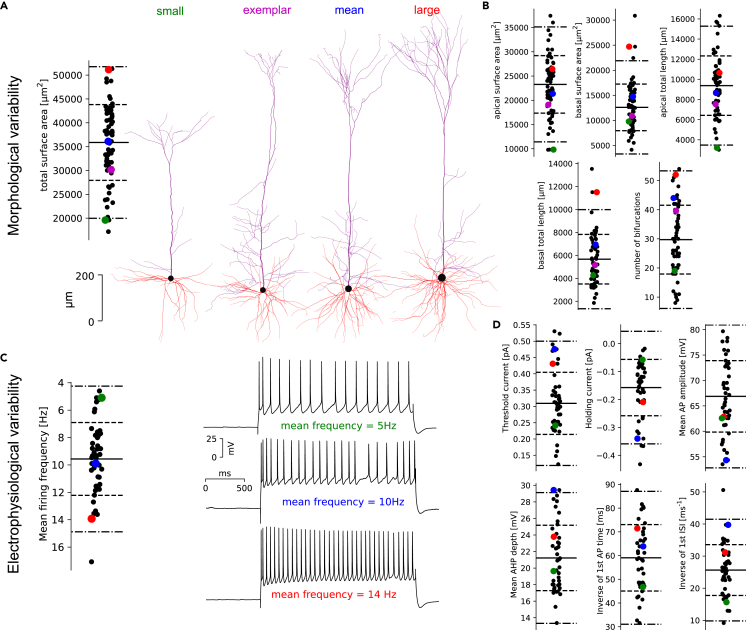
Morphological and electrical variability in thick-tufted L5 pyramidal cells (A) Distribution of total surface areas of thick-tufted layer 5 pyramidal morphologies and four examples with small, mean, and large areas as well as exemplar morphology. Colored dots in (A) and in (B) correspond to these morphologies. The thick line is the mean, dash lines are 1sd and dotted-dash lines are 2sd of the data. (B) Distributions of some morphometrics related to morphological size and surface area. Refer to Figure S5 for more morphometrics and correlations between them. (C) Distribution of mean firing frequencies for the 44 recordings of step protocol at 200% threshold current, with three examples, with small, mean, and large mean firing frequencies. Colored dots in (C) and in (D) correspond to these recordings. (D) Distribution of some other electrical features extracted from the same protocol as well as the threshold and holding current at −83 mV. Refer to Figure S5 for more features and correlations between them.

To illustrate the variability present in the morphologies of the thick-tufted L5PC, we plotted representative morphologies of the mean, large, small, and exemplar cells (see Figure 1A) and extracted 11 morphological features per dendritic type (5 shown in Figure 1B, others shown in with the blue data in Figure S5). The first three cells were selected based on their total surface areas, and the exemplar is the cell with the proximal dendritic surface area closest to the median profile of the population (see blue data in STAR methods). We observed that morphological features such as total surface areas, total lengths, or the number of bifurcations shown in Figures 1A and 1B vary from 3-to 5-fold. These large differences within the thick-tufted morphological type show that a single morphology cannot be a faithful representation of the entire population. Nevertheless, we will need to choose one exemplar morphology to sample electrical models with MCMC in the next section. We pick the dendrites of the morphology with the proximal surface area profile closest to the mean profile of the population and create a soma and AIS with the average size from the population (see STAR methods for more details).

To illustrate the variability of electrophysiological properties of L5PCs, we plotted the distribution of some of the 61 extracted features in Figures 1C and 1D (see Table S1 for the complete list of features). Features are extracted from traces obtained during the experimental application of specific protocols,^32^^,^^42^ and we chose as an illustration to use the protocol of 200% rheobase current step. In the example in Figure 1C, we selected three recordings from three cells with different mean frequencies for this step protocol. The mean frequency ranges from 5Hz to 14Hz, a nearly 3-fold range. The variability of other electrical features, such as holding current (required current to hold the cell at −83 mV), threshold current or other firing properties is also large (see Figure 1D).

Overall, a few correlations are observed between morphological features (see blue data in Figure S5), and similarly for electrical features (see blue data in Figure S4). For example, trivial correlations between mean firing frequency with inter-spike intervals (ISI), longer apical dendrites with larger apical surface areas, and less trivial ones such as threshold current with time to first action potential (AP) or mean frequencies across step current amplitudes. These observations suggest that, as is the case for morphological classes,^47^^,^^49^ electrical features are not simply linearly correlated. In the case of morphologies, simple morphometrics cannot sufficiently describe the complexity of branching structures^50^ while for electrical features, the non-linear, voltage, or calcium-dependent dynamics of the ionic channel conductances create a complex interplay between ionic currents.^51^

### MCMC sampling of electrical models

To reproduce the experimental variability of electrical features in our dataset we built multi-compartmental electrical models composed of the examplar morphology and a set of 30 free parameters based on Hodgkin-Huxley mechanisms, as described in Markram et al. and Reva et al.^32^^,^^42^ (see also STAR methods). We then apply the MCMC method to sample electrical models in this parameter space as follows.

First, to assess the validity of a given set of parameters p to reproduce a target neuronal type, we compared the feature values extracted from simulated traces under specific protocols with the mean and standard deviation of the same features on the population of experimental recordings (see STAR methods). More precisely, we computed an absolute *Z* score for each evaluated feature and a global cost function as the largest score across all features. Often, the sum of the scores is used as a cost C(p) to quantify the quality of an electrical model (see Van Geit et al.^52^). Here, instead, we used the maximum score as it results in overall better models by preventing any *Z* score from growing too large relative to the others. Indeed, there could be situations where one feature has a large score, but the optimization algorithm is stuck in a local minimum from where it is unable to improve this feature, it will keep trying to reduce the sum of scores by changing the other features, which are probably already at an acceptable level. With the maximum of the scores, such a local minimum will not be seen at all. In addition, it prevents redundant features to bias the cost in their direction. For example, if the spiking is regular, all the interspike intervals will be highly correlated, hence the sum of scores will contain several times the same information.

From the cost function, we defined a probability function on the parameter space,

(Equation 1) $$P\left(p\right)\propto \exp\;\left(-\frac{C\left(p\right)}{T}\right)\text{,}$$

parameterized by a temperature parameter *T*. For lower *T*, the generated samples remain around local minima of the cost function while for larger *T* the samples cover more volume with larger costs. In the extreme of T→0, we theoretically recover the global minimum, and T→∞ leads to a uniform sampling of parameters. Sampling from our cost function ensures that most models have a low cost and maximal variability as compared to samples extracted during optimizations. Indeed, this statistical method is mathematically guaranteed to produce samples from this distribution. To do so, we ran several MCMC chains from random initial conditions, which we updated using the Metropolis-Hastings algorithm with multi-variate Gaussian prior (see STAR methods). The sampling quality was validated with an acceptance rate above 50% and fast decaying auto-correlation (see Figure S3).

**Figure 2:**
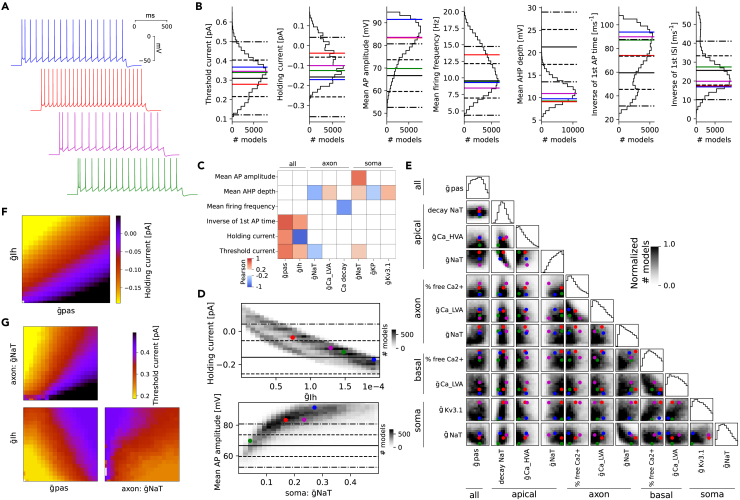
Electrical model generation with MCMC (A) Traces of the step 200% protocol for four models picked such that the red has large AP amplitudes, green has small amplitudes while blue and magenta have amplitudes around the median. We refer to Figure S10 for currentscape plots of these traces. (B) Distribution of some feature values of step 200% protocol for models sampled via MCMC with cost <4. The thick line is the mean, dash lines are 1sd and dotted-dash lines are 2sd of the experimental data (same for all panels). (C) Correlation matrix between features of (B) and parameters mostly correlated with these features (Pearson >0.2 and p value of 0). (D) Density of models for holding current at −83 mV and mean AP amplitude as a function of their mostly correlated parameters, selected from (C) The colored dots represent the four models in (A). (E) Corner plot of model densities with one-dimensional marginals on the diagonal. The gray scales are normalized per pair of parameters and only the most correlated (Pearson >0.2 and p value of 0) pairs of parameters are shown. The colored dots represent the four models in (A). (F) Average holding current at −83 mV value over all parameters except for the two mostly correlated parameters to predict holding current (${\tilde{g}}_{\text{pas}}$ and ${\tilde{g}}_{I_{h}}$). (G) Average threshold current value over all parameters except the three mostly correlated parameters to predict threshold current (${\tilde{g}}_{\text{pas}}$, ${\tilde{g}}_{I_{h}}$, and axonal ${\tilde{g}}_{\text{Na}}$).

Using this MCMC sampling method of the parameter space, we obtained $273^{\prime }088$ models, from which $209^{\prime }653$ have costs below our threshold of 5sd. We checked whether the obtained population of models reproduced the experimental variability of the electrical features (Figures 1C and 1D). Most of the distributions are centered around the experimental mean (solid lines in Figure 2B), except for the mean AP amplitude, inverse time to first AP and mean after-hyperpolarization (AHP) depths (Figure 2B). For mean AP amplitude and inverse time to first AP, as some models are close to the mean experimental value, it could be possible to perform a specific selection for these features to obtain a distribution closer to the experimental population. However, for the mean AHP depth, all models present a smaller value than the mean experimental value, showing a limitation of our modeling approach to reproduce this specific feature.

The large population of models gave us the opportunity to explore the possible causal link between the model parameter values and the feature values. For this reason, we looked at the correlation between a subset of features and parameters (Figure 2C) measured with the Pearson coefficient. We found that some features have a large correlation with few parameters (with up to 0.87 between holding current and ${\tilde{g}}_{I_{h}}$ in Figure 2D (top) or 0.68 for mean AP amplitude and somatic Na in Figure 2D [bottom]). On the contrary, other parameters (such as AHP depth) present low correlations with a large subset of parameters (see AHP depth in Figure 2C). Therefore, AHP depth is controlled by more parameters than other features, making it intrinsically harder to control. While, holding current at −83 mV is highly correlated with ${\tilde{g}}_{I_{h}}$ conductance, (Figure 2D [top]), the variability of this feature cannot be fully explained by the variability in ${\tilde{g}}_{I_{h}}$. The additional variability partially comes from ${\tilde{g}}_{\text{pas}}$, which is also correlated with holding current (Figure 2F). In general, the variability of feature values depends on the interplay between several underlying parameters, and more parameters are required for features related to spiking (Figures 2G and S9).

This study of the causal link between parameters and features allows a refinement of the MCMC sampling. For example, the correlation between the mean AP amplitude and the maximum conductance of the somatic sodium channel (${\tilde{g}}_{\text{Na}}$) Figure 2D (bottom) suggests a way to control the AP amplitude by adjusting the upper bound of the somatic ${\tilde{g}}_{\text{Na}}$ to around 0.15. In this way, most models will be more centered around the experimental mean (around 68 mV) during MCMC sampling, thus producing more valid models for the same computational cost than sub-sampling. We also looked if constraining the models below 3sd for all the features produces the emergence of correlations between parameters (Figure 2E). For example, we observed a negative correlation between maximal somatic Na conductances and maximal axonal Na conductances (as already noticed in Schneider et al.^53^). This correlation is partly imposed by the constraint from the AP amplitude. In fact, if the somatic Na conductance is high, the axonal conductance has to be low to maintain AP amplitude within the experimental range. This representation of the accepted models (constrained by the cost) can be seen as a global map of the parameter space where valid models are located (with costs below 5sd). Regardless of their locations, the models will globally perform equally well (see Figures 2A and 2B) but will contain subtle differences that can be quantified or controlled with MCMC sampling.

### Generalization of electrical models to a population of morphologies

To sample valid models with MCMC we used a single exemplar morphology assumed to represent an entire population of morphologies. Since our original motivation was to obtain a population of models and morphologies such that any pairs were valid with high probability, we needed to ensure that our MCMC models remained valid on the entire population of morphologies. First, to check the validity of models, we used a cost function based on a reduced set of features, discarding features based on backpropagating action potentials (bAP), which are sensitive to the shape and length of the dendrite tree. Then, due to the large number of models obtained from MCMC, we began by randomly sampling 100 models with a cost below 3sd (out of $10^{\prime }678$), such that the MCMC density of models was preserved. This results in models being more likely to be away from the region of invalid models in the parameter space, hence possibly more generalizable. Indeed, one expects that if a model is close to the boundary of this region, a change in the morphology would affect the effective conductances of the whole cell, which may bring a cell out of the valid region.

To improve the generalizability of our electrical models on various morphologies, we adapted the soma and AIS surface area by computing the relative input resistances between soma, AIS, and dendrites. Indeed, as it was noticed in early works such as,^54^ or more recently in,^42^^,^^45^ the relative input resistances between the AIS, soma and dendrites are essential to determine the excitability of the cell. The important quantities to consider in this context are called ρ factors^54^ and are defined as the ratio of input resistances of specific compartments as

(Equation 2) $$\rho =\frac{R_{\text{in},\text{soma}}}{R_{\text{in},\text{no}\;\text{soma}}},\rho_{\text{AIS}}=\frac{R_{\text{in},\text{AIS}}}{R_{\text{in},\text{no}\;\text{AIS}}}$$

where $R_{\text{in},\text{soma}}$ and $R_{\text{in},\text{AIS}}$ are the input resistance of the isolated soma and AIS compartments, and $R_{\text{in},\text{no}\;\text{soma}}$ is the input resistance of all the neurites at the soma location but without the soma, so effectively recorded at the beginning of the AIS. Similarly, $R_{\text{in},\text{no}\;\text{AIS}}$ is the input resistance of the soma and dendrites, without the AIS, recorded at the soma. In, Hay et al.^45^ the authors rescaled the maximal conductances of the AIS and soma to match the ρ factors of the optimized cell. Here, we consider that the AIS and soma sizes have variability that can be exploited to improve the generalization of fixed electrical models. We thus rescaled the surface area of the AIS and soma of each morphology such that the ρ factors match a given target value. We had to find the target values for ρ and $\rho_{\text{AIS}}$ for each pair between the 100 electrical models and the 4 examplar morphologies, one for each morphological type. The target ρ values are found with a grid search on AIS and soma scales for the optimal cost (see STAR methods). We thus have one set of the target ρ factors per model and morphological type.

**Figure 3:**
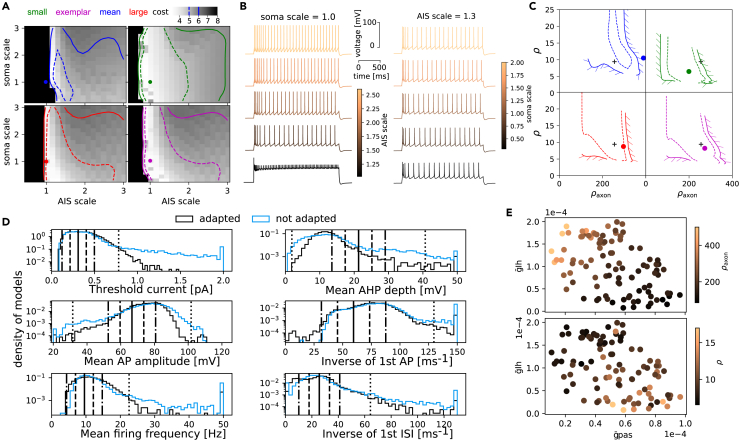
Generalization with AIS/soma adaptation (A) Cost of models by varying soma and AIS size for the four morphologies of Figure 1A. The region enclosed in dashed lines has a cost below 5sd while that in solid lines has a cost below 6sd. The dot shows the location of the cell with unscaled AIS and soma. (B) Step traces of the large morphology with fixed soma (left) and AIS (right) scales but with varying AIS (left) and soma (right) scales. (C) The model validity region with cost of 5sd (dashed) and 6sd (solid) of panel A is mapped to the ρ factor plane. The dots again represent the unit AIS and soma scales while the black cross is the location of the best model obtained by minimizing the cost from the exemplar with the grid search in (A), bottom right. (D) Histograms of feature values on all pairs of electrical models and morphologies with (black) and without (blue) soma and AIS adaptation. The thick line is the mean, dash lines are 1sd and dotted-dash lines are 2sd of the experimental data. (E) ρ and $\rho_{\text{axon}}$ as a function of the ${\tilde{g}}_{\text{pas}}$ and ${\tilde{g}}_{I_{h}}$ parameters.

We first show how rescaling the AIS and soma size changes the cost of a model (Figure 3A) on the four morphologies shown in Figure 1. We observe that for all morphologies, a reduction of the AIS size leads to a large increase in cost (clipped at 8sd) while the change of soma size does not impact the firing pattern (Figure 3B). This result is related to the coupling between axonal and somatic compartments, as recently studied in more detail with a similar sampling approach in Zang et al.^55^ The exact shape of the level set of cells with costs less than 5sd largely depends on the morphology (Figure 3A, the region below 5sd is enclosed in the dashed lines). For some morphologies, the original AIS and soma size do not produce a valid model (Figure 3A, color dots). In particular, the region of valid models is small for large cells but large for small cells. By converting the AIS and soma scales to ρ factors (Figure 3C) we confirm that the target ρ factors (black cross) obtained from the exemplar cell are within, or close to the validity region of other morphologies. If the cells are too different, such as our small and large morphologies (see Figure 1A), their validity region in the ρ factor plane may not overlap for both to work with a single target ρ factor. As this overlap depends on the electrical model, it defines its generalizability on a population of morphology (see next section).

For each model, we obtained target ρ factors using the examplar morphologies and we used the targets to fit the AIS and soma size of each morphology (see STAR methods). These adapted cells have a clear reduction of extreme values of features as compared to non-adapted cells (Figure 3D), and in particular for threshold current, where 7.4% of the cells have a threshold above 5sd without adaptation while only 1.1% of cells are above 5sd after adaptation. As ρ factors are based on input resistance, they are highly sensitive to the passive leak and $I_{h}$ channel densities (Figure 3E). It should therefore be possible to predict the target ρ values directly by looking at these parameters instead of computing the AIS and soma scaling on the examplar morphologies. To demonstrate it, we fitted a regressor learning model (see STAR methods) to predict the values of ρ factors from the model parameters and achieve a 10-fold accuracy for the prediction of ρ of 1.36±0.34 and for $\rho_{\text{AIS}}$ of 27.5±8.6. As described in the next section, this AIS/soma adjustment, which corresponds to some artificially controlled variability, is important to ensure that a model can be generalized to a population of morphologies with substantial variabilities.

### Morpho-electrical selection and variability

**Figure 4:**
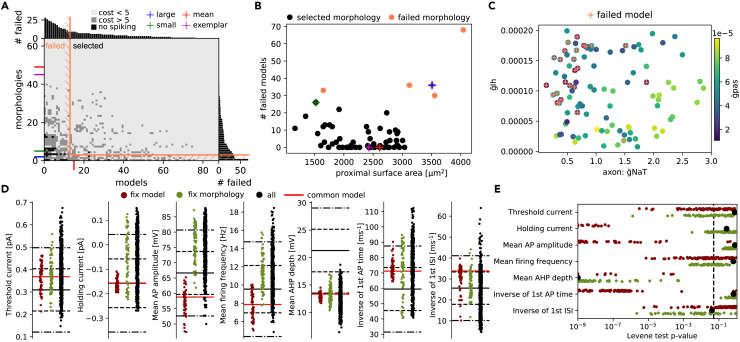
Morpho-electrical selection and variability (A) Selection matrix for thick-tufted cells where selected models and morphologies are delimited with the orange line. Each pixel corresponds to a model, where models in light gray have scores below 5sd, in gray have some features with costs higher than 5sd and in black pixels are models that do not spike. The four morphologies of Figure 1 are represented with colored ticks, and the model of Figure 4 with a red tick. We refer to Figure S11 for the same selection matrix without AIS and soma adaptation. (B) Proximal (up to 500μm) dendritic surface areas for all morphologies with non-selected morphologies in orange versus the number of failed models for each morphology. The four morphologies are also highlighted with colored crosses. (C) From the classification of selected models from their parameters with a classifier algorithm (10-fold accuracy of 0.89±0.08), three parameters are most important, with a clear correlation. Models with small ${\tilde{g}}_{\text{pas}}$ values also contain models for which we have not been able to fit the AIS/soma resistance model (not shown in A). (D) Distribution of features obtained by freezing the red model (57 points in red), the exemplar morphology (75 points in green), or 500 models randomly sampled from the 5197 total pairs with cost <5 (in black). The red line corresponds to the value of the black model with exemplar morphology. The thick line is the mean, dash lines are 1sd and dotted-dash lines are 2sd of the experimental data. (E**)** p value of Levene’s test centered with mean. Each dot is the p value of this test between the experimental data shown in Figure 1 and all MCMC models with cost <3 in black, subsets of models in red and morphologies in green. The distributions per feature in (D) correspond to single points in this plot.

Even with AIS and soma adaptation, it is not guaranteed that all pairs of models and morphologies will work together. For example, the morphology with the smallest area (green color Figure 3C) is close to the non-valid regime. It is possible that a better choice of the target ρ factors exists, but given our current algorithm based on a single exemplar morphology, the success rate for model morphology pairs is satisfactory, as shown in Figure 4A. We distinguish three levels of models: valid models (light gray), models for which there are some features with scores larger than 5sd (gray) and models where the search for threshold current failed, hence they do not spike during step protocols (black) (Figure 4A). Only the first class of models with all scores below 5sd will be considered as models representative of the cell type. By sorting the electrical models from the less generalizable (failing when applied to a lot of morphologies) to the most generalizable (produce good cells for all the morphologies), we were able to select a large fraction of morphologies and electrical models (delimited with orange lines) such that most pairs have a cost below 5sd. In Figure S11A, we show the same selection matrix without adaptation, which needs a more drastic selection of models and morphologies. Models that do not generalize well on the morphological population could also be seen as less robust under morphological perturbations than others. A similar recent study, but with perturbation of ion channel maximal conductances was done in,^55^ which found that the coupling between somatic and axonal compartments was important to maintain the rebound bursting features. Here, this coupling is leveraged to adapt the AIS and soma scales with the ρ factor.

Given the morphological variability of our population of pyramidal cells, we can investigate the relationships between some global morphometrics and generalizable cell models. For example, the proximal (up to 500μm) surface areas of selected (black) and non-selected (orange) morphologies is a good predictor of the number of failed models, of model generalizability (see Figure 4B). In fact, best morphologies (i.e., that failed with a small number of electrical models) have surface areas close to the exemplar morphology, while most distant morphologies (with large or small surface areas) are much less generalizable (they failed with a large number of electrical models). This result indicates the range of proximal surface areas of morphologies that can be generalizable from a single exemplar morphology. We also show the same result without adaptation where the region of validity is below the exemplar (see Figure S11B). If the largest cells are important for a given study, two choices are possible. One can recalibrate the ρ factors using a larger exemplar morphology, or, if this is not sufficient, run MCMC sampling again to produce another set of models. The second choice will arise only for extreme morphological differences where the valid regime in the parameter space is not reached with the MCMC sampling based on the first exemplar.

In this context, we ask whether it is possible to predict if an electrical model is generalizable from the parameter values. From inspecting parameter distribution between each set of models (generalizable and not generalizable) there are no evident differences (not shown), but when training the XGBoost classifier^56^ (see STAR methods), the 10-fold accuracy reaches 0.89±0.08, showing that it is possible to accurately predict the generalizability of a model on a given population, but only via nonlinear, higher-order combinations of parameters. In Figure 4C, we show the values of the main parameters detected via Shap feature importance analysis^57^ involved in predicting the generalizability of models on the population of morphologies. We found that small passive and axonal Na conductance and large $I_{h}$ conductances are more likely to produce non-generalizable models.

Finally, from this population of models and morphologies, we assessed how well they match the variability of the experimental data (Figure 1), and in particular, the morphological or electrophysiological variability alone is sufficient to reproduce the experimental variability. For this, we fixed the model used in Figure 4 and compared the distributions of the features when evaluated on all selected morphologies (Figure 4D, red dots). We found that, for this specific electrical model, the morphological variability is not sufficient to explain the experimental features variability. In fact, we can see that for features such as holding current, AP amplitude or time to first AP, distributions of features obtained by modification of morphology are less variable than experimental features (Figure 4D, the thick line is mean, the dashed line is 1 sd, and the dotted dashed line is 2sd of experimental data). Then, we fixed the exemplar morphology and compared the distributions of the features when evaluated on all selected electrical models (Figure 4D, green dots). Fixing a morphology seems to produce more consistent variabilities across features, except for AHP depth, which is biased toward low values already in the MCMC sampling. Therefore, the experimental variability of electrical features seems to mostly arise from the variability of the ion channel densities in the population. We finally looked at the feature distributions when testing all the pairs between selected morphologies and selected electrical models (Figure 4D, black dots) and found an even larger feature variability, showing that combining both morphological and electrophysiological variability is important to reproduce experimental variability. In order to quantify these variabilities, we compared these distributions with the experimental data with the Levene statistical test (centered with mean) (Figure 4E) by fixing all morphologies (green) or all models (red). We found with this procedure, more feature distributions presented a p value for the Levene-test smaller than 0.05 with a fixed electrical model than with a fixed morphology, consistent with the results in panel d. Finally, the black dots show the comparison between experimental feature distributions and distributions when testing all the 5197 pairs between electrical models and morphologies which all have p-values above 0.05 (distributions not distinguishable from the experimental one) but for the mean AHP depth. Therefore, applying MCMC sampling, soma and AIS scaling by ρ factors procedure and selection of generalizable electrical models and morphologies, allowed us to build a population of models that reproduces the variability of the features found in a neuronal population.

### Further generalizations

From MCMC sampling, we only selected 100 models to perform AIS/soma adaptation to create a set of valid pairs of electrical models and reconstructions. This was primarily due to the computational cost of calibrating the ρ factors and evaluating all the models on morphologies to select them. By using standard tree-based machine learning regressors and classifiers (see STAR methods) we fitted models for the ρ factors, the AIS/soma resistances, and model generalizability, we were able to use more models from MCMC sampling with reduced computational cost. In total, we evaluated 13794 new pairs of selected morphologies and sampled models where 92.8% have cost below 5sd (as light gray pixels in Figure 4A) and 98.9% that are able to fire (as gray pixels in Figure 4A). Once calibrated, our method of adapting the soma and AIS can be applied to models sampled with MCMC not yet used and produce a larger number of generalizable models without the need for expensive calibration and validation of ρ factors and fully leverage the variability created by MCMC sampling for statistical analysis or circuit building.

Increasing the number of models is often not sufficient to capture the entire biological variability, especially if experiments involve inter-neuron connectivity or synaptic inputs. For this, one could use more reconstructions if they fall within the estimated valid surface area bounds, or leverage neuronal synthesis algorithms. Here, we generated morphologies with the algorithm of Kanari et al.^49^ based on the topological descriptor introduced in Kanari et al.^50^ (see STAR methods) to show that if morphologies fall within the original population, there is a high probability that they will perform well on all selected models. We found that 94.8% of the generated pairs from 100 synthesized morphologies had a cost below 5sd, but only a few with small oblique surface areas were consistently failing. This suggests that some specific morphological features should also be taken into account to refine how morphologies are classified to produce consistent populations for electrical modeling.

In this work, we focused exclusively on layer 5 pyramidal cAD cells, but in Figure S8 and STAR methods, we show the application of MCMC and generalization on another electrical type, the continuous non-accommodating interneuronal types. We found similar properties to the cADpyr models, whereby MCMC captures non-trivial parameter correlations, small morphologies are harder to generalize but from the selected set of morphologies and models, we are able to generate more valid models. Our approach of MCMC sampling and further model and morphology selection thus works on any electrical types, provided sufficient features are available and can be used to construct models with controlled variability for several electrical types, for inter-type comparisons, or building more realistic microcircuits.

## Discussion

In this work, we present a framework to study detailed biophysical models of neurons. In particular, we take into account the experimental variability of morphologies and electrophysiological features within cell types. For this, we leveraged a standard statistical method, MCMC, to generate thousands of electrical models reproducing experimental variability, and we generalized them to a population of detailed reconstructions of morphologies by adjusting AIS and soma size according to calibrated ρ factors.

With this approach, we produce a population of models with feature variability close to experimental data and we demonstrate that morphological variability alone is not sufficient to reproduce the observed electrical behavior. The variability of electrical models as measured by the feature values is a direct consequence of the parameter variability.^9^ In order to ensure that the feature variability matches the experimental data, strong constraints are necessary on the model parameter space, which is possible to be analyzed with MCMC samples. The study of these constraints is out of the scope in the current paper, but preliminary analysis indicates that a few constraints are low-dimensional, while many are high-dimensional, in particular for complex features such as average AHP depth during a step protocol. These constraints on the parameter space are a result of the choice of protocols and features. Thus to obtain more precise models and reproduce specific firing properties, such as BAC,^58^^,^^59^ even stronger constraints are most certainly required, for example, more specific apical ion channels. Hence, such MCMC sampling methods, or improvements of them,^43^ provide a tool to detect model limitations and investigate their origins.

The study of model generalizability on a population of morphologies suggests a new morphological grouping based on specific morphological features, related to their electrical activity. Given an exemplar with calibrated ρ factors, we find that the proximal dendritic surface area (i.e., up to around 500μm including basal and oblique dendrites) is a good predictor for the validity of the morphology on that model. However, other more specific morphological features, such as oblique dendrite areas, also have an impact, as was discovered with synthesized morphologies in STAR methods. As such morphological features do not necessarily correlate with more generic classifications involving specific apical features (tuft, no-tuft) or axonal ones (mostly for inter-neurons), it may be possible to create more targeted morphological types of neurons based on these to ensure that all morphologies of a specific type will perform well with a single set of ρ factors. In addition, it may be interesting to study what fundamental properties of a model ensure its generalizability. For example, Otopalik et al.^60^ showed that some electrophysiological features of gastric mill neurons in crustacean stomatogastric ganglion are not affected substantially by the morphological anatomy of the cells.

The sampling of large parameter space to study neuronal models is an active research topic, with several recent advances^31^^,^^44^^,^^61^^,^^62^^,^^63^^,^^64^ including machine learning for Bayesian inference of model parameters in the context of single cells or even circuit simulations. We instead provide an alternative framework to sample models under experimental constraints without using machine learning. For machine learning algorithms, a sufficient number of samples are needed to obtain reliable models, whereas in our case, only a few samples could be reliably sampled to fit the experimental data. Our MCMC method is thus more appropriate for some use cases, such as model building (adjusting parameter bounds for example) or generalization on a population of morphologies (for which many samples will be too computationally intensive), but can still produce many models for finer statistical analysis. Our method lacks some of the predictive power of machine learning models for regimes not characterized by the electrical features, but with large enough sampling and specific studies of a subset of models, some predictions could also be made.

Overall, this work proposes a new perspective on building detailed electrical models of neurons with Bayesian statistics via the MCMC method. We show that we can unravel subtle mechanisms via the study of parameter and electrical feature correlations while providing a consistent framework to assess the quality of electrical models by controlling their variability on a single morphology or for a population with its own morphological variability. This work opens further research avenues to study the interplay between electrical models and morphologies, co-regulation,^65^ energy efficiency,^66^^,^^67^ the impact of morpho-electro variability in circuit simulations,^9^^,^^68^^,^^69^^,^^70^ degeneracy^27^^,^^28^^,^^29^ or links with gene expressions.^64^ In addition, MCMC sampling of electrical models may become instrumental in making progress on the long-standing question of redundancy and synergy in biology, and in particular, in neurons.^10^^,^^51^

### Limitations of the study

This study also had several limitations. The quality and more importantly the consistency of the recording and neuronal reconstructions may affect significantly the feature values used to constrain MCMC sampling. We did not study the robustness of the sampling with respect to the experimental data accuracy by, for example, adding or removing data points randomly. In order to quantitatively use the measured parameter correlations from the sampling, one must ensure that they do not depend significantly on the inaccuracies of the experimental data. Then, in order to obtain good coverage of large parameter space, many samples are needed, and, depending on the computational cost of evaluating a single model, this can quickly become unfeasible on small computers. In these cases, a tradeoff has to be made between the quality of the sampling and the computational cost. It may be possible to implement more modern and faster converging MCMC methods to improve on this aspect.

## STAR★Methods

### Key resources table

**Table:** 

REAGENT or RESOURCE	SOURCE	IDENTIFIER
**Deposited data**		

Data and script to reproduce figures	This paper	https://doi.org/10.7910/DVN/FGWMUF
Morphological data	Kanari et al.^49^	https://doi.org/10.5281/zenodo.5909613
Electrophysiological data	Reva et al.^42^	https://doi.org/10.5281/zenodo.8220287

**Software and algorithms**		

emodel-generalisation	This paper	https://doi.org/10.5281/zenodo.8269363
BluePyOpt	Van Geit et al.^52^	https://doi.org/10.5281/zenodo.8136124
NEURON	Hines et al.^71^	https://www.neuron.yale.edu
Dask	Dask Core Developpers	https://www.dask.org

### Resource availability

#### Lead contact

Further information and requests for resources and code should be directed to and will be fulfilled by the lead contact, Alexis Arnaudon (alexis.arnaudon@epfl.ch).

#### Materials availability

This study did not generate new unique reagents.

### Method details

#### Morphology dataset

We use the layer 5 pyramidal morphology dataset of,^42^^,^^46^ which is an extended version of the one from.^32^ It consists of four subtypes, with 64 thick-tufted cells, 38 bitufted cells, 30 small tufted cells and 27 un-tufted cells as defined in.^47^

#### Diametrization algorithm

The input to the algorithm is a population of morphologies, and it will be applied to a single type of neurite (basal and apical). For each section of morphology, we compute the path distance from its end to the downstream terminal point that is furthest away (see Figure S2A). We then normalized these values by the largest path length of the morphology. We also record the section mean diameters and fit a polynomial function of diameter as a function of the distance, which will be our diameter model, see Figure S1E.

The diametrization of a given morphology then first uses this model to assign a diameter to each section independently. It then introduces a linear tapering of diameters along each section such that the first diameter is the assigned one, and the last is the average between the first of the current section and the largest first diameter of child sections. If the section is a terminal section, we read the last diameter from the model.

In Figure S1B we show two examples of rediametrized morphologies, where the left morphology has similar diameters, and the right one has reduced diameters. In Figure S1D we show the changes of surface areas induced by the rediametrization, mostly affecting the large surface areas (such as for the right morphology), but overall making the distribution of dendritic surface more narrow.

This algorithm is not based on any biological principle, such as for example,^48^ but resulted in consistent dendritic diameter profiles so that the possible artifacts of the reconstructed diameters did not bias our generalization results, but only the morphological shapes did. More work could be done in assessing the impact of diameters as a function of distance to the soma for example.

The diametrization algorithm is implemented in the open-source python package https://github.com/BlueBrain/diameter-synthesis.

#### Exemplar morphology

From a population of morphologies, we select an exemplar morphology as follows.

We compute the average surface area of the soma with the NEURON simulator for greater consistency with Neurolucia format in Figure S2A as well as the average radius in Figure S2B. We then create a single cylindrical compartment with an average radius and length computed such that the surface area is also the average from the population.

We extract the diameters of the first 60μm of the reconstructed axons (see Figure S2C), assumed to be the AIS of the neuron and use the average diameter to create a two-compartment model of the AIS with constant diameter. We ignore the tapering near the soma, which does not affect significantly the electrical features (not shown). We then discard the reconstructed axons as we do not electrically model them in detail but replace them with a constant diameter AIS of length 60μm followed by a 1 mm myelinated section. The AIS will serve to generate action potentials, and the myelinated section will act as a sink, as an approximation of the effect of removed axonal branches.

In addition, we need to select a morphology that is most representative of the population. As most experimental protocols and recordings we will use are somatic, electrical models will be most sensitive to the proximal surface area of dendrites. We compute the total surface area of all dendrites as a function of path distances in Figure S2D, compute the median profile and select the morphology closest to use as exemplar dendrites. We apply this procedure to construct a global exemplar morphology from all L5 PC cells which we will use for MCMC sampling. We also create m-type specific exemplars where the dendrites are selected using only morphologies within this m-type, for ρ factor calibrations.

#### Electrical models and cost function

We use the model of,^42^ based on the ones from.^32^ They are composed of a set of coupled nonlinear equations assigned to each compartment in a morphology. For each compartment, a subset of these equations is parametrized by properties such as radius and lengths and coupled to the adjacent compartment via current conservation condition.^71^ All dynamical equations are of the form

(Equation 3) $$\frac{du}{dt}=-\sum_{k}I_{k}\left(u,t\right)+I\left(t\right)\text{,}$$

where *u* is the membrane potential, I(t) the applied current and $I_{k}$ the various ionic currents indexed by *k*, non-linearly depending on the voltage.

Several protocols defining I(t) are applied on the soma and recorded in the soma or along specific dendrites. These protocols are the same as in,^42^ where the most important are the step protocols with amplitude relative to the rheobase of the cell. The rheobase is found with a bisection search, where the lower bound is the holding current, defined as the current to hold a cell at −83 mV and the current to be at −30mV, estimated from the input resistance. The holding current is also found from a bisection search, which, as for the threshold, is terminated once a certain accuracy on the current is reached (difference between the last upper and lower bound). If the threshold current is above −30mV, we assume that the cell cannot spike, thus subsequent protocols are not evaluated, and the cost of the model is maximal. From these voltages (or other ion channels) recordings, a number of features, such as mean AP amplitude and AP frequency are extracted, as in.^42^

Only a subset of all features is used for analysis steps after performing MCMC. In particular, we discard bAP features, which are too sensitive to apical diameters, and APWaveform where the second AP may not happen due to too short step protocol, SpikeRec because it is related to recovery after a spike, which we do not consider here and IV.

Denoting the simulated *k* feature values as a vector $f=\left(f_{0},\ldots ,f_{k}\right)$, the parameters as $p=\left(p_{0},\ldots ,p_{n}\right)$ and construct a cost function to measure the model quality as

(Equation 4) $$C\left(p\right)=\max_{i}\frac{\left|f_{i}\left(p\right)-\bar{f_{\exp,i}}\right|}{\sigma \left(f_{\exp,i}\right)}=\max_{i}z_{i}\left(p\right)\text{,}$$

where $z_{i}$ are the absolute z-scores of each feature indexed by *i*. Notice that we define the cost as the max of the scores, which is stronger than the sum of the score usually used in optimisation.^52^ In addition, each parameter is assigned a predefined range of possible values, possibly consistent with biological data if any are available.

#### 0.1 MCMC sampling of models

All parameters have normalized versions denoted by $\hat{p}$, such that the available range is [−1,1], and no bias is induced by the various possible units or bound sizes of each parameter. The parameter space for a valid electrical model is thus a subspace Ω of the hypercube ${\left[-1,1\right]}^{n}$ defined as p∈Ω if and only if $C\left(p\right)<C^{*}$, where $C^{*}$ is a maximum cost to consider a model valid.

To generate many set of parameters to cover this set Ω, we use the MCMC method with the Metropolis-Hastings algorithm as follow. We define a probability distribution from the cost function as

(Equation 5) $$P\left(p\right)\propto \exp\;\left(-\frac{C\left(p\right)}{T}\right)\text{,}$$

where *T* is a temperature parameter. The goal of MCMC is to sample from this distribution. For this, we use a normal prior distribution $\pi \left(\hat{p}\right)=N{\left(\hat{p},\epsilon \right)}^{n}$ with variance ϵ. Because the parameter space is a hypercube, if the proposed set of parameters lies outside, we re-sample until we get a point inside the hypercube. For each chain, we then sample a random point ${\hat{p}}_{0}$ in the hypercube, and compute the next point in the chain using the Metropolis-Hastings algorithm:

1. propose i${\hat{p}}_{i+1}∼\pi \left({\hat{p}}_{i}\right)$,
2. draw 1a∼U(0,),
3. accept ${\hat{p}}_{i+1}$ if $\frac{P\left({\overset{⌢}{p}}_{i+1}\right)}{P\left({\overset{⌢}{p}}_{i}\right)}\geq a$, else reject and set i${\hat{p}}_{i+1}={\hat{p}}_{i}$,
4. repeat until the number of iterations is attained.

In practice, we launch several chains for a short so-called burn-in phase with a temperature of 5 and a prior of 0.2sd so that enough chains have reached the valid region Ω. We then restart new chains from a selection of best models obtained in the burn-in phase with a temperature of 0.5 and a prior of 0.02sd, see Figure S11A. To obtain our final set of parameters in Ω, we remove samples with $C>C^{*}$. The parameter ϵ should be set such that the acceptance rate is around 60−80% depending on the chain, to ensure we optimally explore P(p) correctly. The convergence plot in Figure S3A shows the burn-in phase and the longer run, with a uniform sampling of cost values across iterations. In addition, the auto-correlation plot for this run in Figure S3B shows fast decay of correlations after less than 50 iterations, which gives an indication of the minimum number of steps one should due to have a good sampling In Figure S3C, we show for each feature the fraction of time it is the largest, so it defines the cost. A more uniform distribution shows that no features are primarily blocking the chains from reaching low-cost values.

Overall, MCMC sampling can also be used as a tool to improve electrical models. Indeed, the corner plots and one-dimensional marginals such as shown in Figure 2E are useful for adjusting parameter bounds. If many good models are near the upper bound of a parameter, one could increase it (if the value remains meaningful) to maybe obtain even better models. On the contrary, if the distribution is very narrow toward small values, reducing the bounds will improve the acceptance rate of the MCMC sampling. The study of correlations between features (see STAR methods) may also be of interest to detecting the possible lack of feature parametrisability of the model, resulting in a limitation of the lowest possible scores achievable. With the addition of plots such as Figure S3C which indicates which features saturate the cost more often, or even with plots of traces for some specific models, MCMC sampling can be effectively used to propose improvements on the choice of ion channel mechanisms or associated parameter bounds.

#### Correlations of features

In Figure S5A, we compare the feature correlation computed with mutual information between experimental data (see Figure 1) and MCMC sampling (see Figure 2) showing a good agreement. For example, the inter-spike interval correlation is stronger in MCMC than in the data, possibly due to experimental noise not present in our simulations. The correlation between mean frequencies is also higher in MCMC sampling, thus IF curves will have more consistent slopes among MCMC models than experimental data. In Figure S5B we show some of these correlations via scatterplots of experimental and numerical data. In these plots, some experimental outliers (crosses) were detected and removed to compute the mutual information, as they biased the results substantially. It may therefore important to perform such analysis of the experimental data to detect any possible outliers that may not be visible in one-dimensional distributions. In addition, these outliers are few but correspond to stuck cells, thus having stuck cells in the model may be allowed in small numbers, from a pure data point of view.

In Figure S5, we perform a similar analysis but on morphological features of reconstructed and synthesized morphologies (see STAR methods). The pairwise correlations of the selected feature show a good agreement between both sets of morphologies, where only a few features are strongly correlated.

#### Soma and AIS input resistance models

To adapt the size of soma and AIS according to the ρ factors, which are based on input resistances, we need to model the input resistance of isolated soma and AIS as a function of their sizes. For each model, we evaluate the input resistances as a function of the soma and AIS scales from 0.1 to 10 and perform a polynomial fit of order 3 on this data in log-log see Figures S6A and S6B, resulting in four parameters per electrical model for both the AIS and soma. In Figures S6C–S6H we show the parameters mostly correlated with the first three fit parameters (the fourth is also correlated with ${\tilde{g}}_{Kv3.1}$), found by searching for model parameters with Pearson above 0.7 with the fit parameters. We remark that the passive currents control the affine part of the input resistances of these compartments, while the Kv3.1 current controls its deviation slope and possible deviations from a linear relation.

Sometimes, the computation of these input resistance models fails, either due to simulation issues, or poor polynomial fits. In this case, we discard the electrical model for later use. These cases happened more frequently for low models with low passive conductances, where other channels have more prominent effects, making the input resistance relation with the AIS and soma size more complex.

#### M-type specific rho factors

To find optimal ρ factors we use the exemplar morphology for each m-type and scan for AIS and soma scales from 0.5 to 1.5 in 10 steps and smooth the scores (with reduced feature set) with a Gaussian kernel of 0.1 width, to obtain a matrix such as in Figure 3, but with lower resolution for computational efficiency. The point of the lowest score is selected as the target scale for each pair of m-type/electrical models (as the black cross in Figure 3C), which are then converted to ρ factors and stored to later adapt the AIS/soma.

#### AIS/soma adaptation algorithm

To adapt the AIS and soma size to match the target ρ factors estimated in STAR methods, we use an iterative algorithm as follows. First, we adapt the AIS size after evaluating the input resistance of the dendrites and soma and using the polynomial fit of the AIS resistance model to assign an AIS scale. We then do the same for the soma, with input resistance computed with dendrites and scaled AIS. We repeat this two times to ensure that the scales of AIS and soma have converged. We do not require a precise convergence to the target rho factor because it has been calibrated with a small resolution for computational efficiency (see STAR methods) and nearby values are also likely to work equally well. Nevertheless, this two-step algorithm converges to ρ factors values at around a few per cent of the target.

#### Further generalization with machine learning classifiers and regressors

Due to the computational cost of searching for target ρ factors for each electrical model and fitting input resistance models of the AIS and soma, we cannot use too many sampled models from MCMC. Hence, to further expand the pool of models, we leverage machine learning algorithms to estimate these values.

For that, we use gradient boosting tree-based learning algorithm XBGboost^56^ with the classifier or regressor and default parameter from the Python implementation. We used default parameters and a learning rate of 0.1 and reported the accuracies computed with 10-fold validations with 5 randomized repeats.

First, to learn a model of input resistances, we apply the XGBoost regressor on the normalized model parameters with a Pearson correlation larger than 0.7 (shown in Figures S6C–S6H), to prevent any overfitting. If no parameters are correlated enough, we replace the model with the mean value of this parameter. On a new set of models, we then evaluate these models to estimate the input resistance polynomials for both AIS and soma. To prevent our ML model from extrapolating the values, we sub-sample models so that their parameters are between the 10’s and 90’s percentile of the trained set (black lines in Figures S6C–S6H).

We train the same regressor model to predict the ρ factors with normalized model parameters that have a Pearson correlation larger than 0.4 with the ρ factors. The choice for this lower correlation is from the fact that the calibration of the ρ factors is based on a coarser scan of the parameter space, hence it is noisier and we do not expect very high correlations. Again, if no parameters are correlated enough, we replace the model with the mean value of this parameter. One the additional models, we again use these ML models to predict their ρ factors, but only if their parameters land between the 10’s and 90’s percentile.

Finally, we use the XGBoost classifier with the same parameters as previously to estimate electrical model generalization from their parameter values. Using the Shap feature important analysis,^57^ we could find the most important parameters to predict the model’s generalization, shown in Figure 4C. We then used only models that this classifier predicts as generalizable to test whether our ML calibration produces valid models (see STAR methods). We used all normalized model parameters to train this classifier, likely overfitting the results, but without much impact.

#### Increasing morphological variability with neuronal synthesis

In addition to generating many electrical models of a given cell type reproducing experimental data, we can generate morphologies reproducing experimental data for a morphological type. This can be done with neuronal synthesis algorithms trained from the selected population of reconstructions. Several algorithms are available, such as^72^^,^^72^^,^^73^ but we will use here the more recent, topologically based algorithm of.^49^ We generated 100 thick-tufted morphologies for which we adapted the AIS and soma scales for each selected model, and evaluated all pairs of synthesized morphologies and models.

First, in Figure S3, we confirm from^49^ that the experimental morphological variability is well reproduced and in particular the correlations between some main morphometrics. After the evaluation, we find that 94.8% of the pairs of morphologies and models have cost below 5sd and 98.7 have cost below 10sd. In Figure S7, we show a more detailed analysis of this result, and in particular only 3 morphologies are responsible for a large part of the costs above 10sd. After further inspections, we found that the surface area of the oblique dendrites is a good predictor of the failure of the morphology on many models. We leave a more detailed analysis of the reasons for such a correlation for future works, but it shows that some specific aspects of the branching structure may matter for electrical modeling, even when measured at the soma.

#### cNAC electrical model

To show that the MCMC methodology and results also apply to other electrical models, we used the continuous non-accommodating (cNAC) electrical model of.^32^^,^^42^

In Figure S8 we show the main results of MCMC and generalization on Martinotti cells. The exemplar was chosen based on m-types with most reconstructions, with a total of 191 interneurons and 6 morphological types (L23_LBC, L5_MC, L4_LBC, L23_MC, L1_HAC and L4_NBC). The exemplar for MCMC (Figure S8A) was an L23_MC (Figure S8B, left) and we illustrate in Figures S8C and S8D the generalization on L5_MC cells.

We also applied the generalization procedure with ML models of ρ factors and AIS/soma input resistance to obtain 87.1% of the 7620 evaluated pairs with a score below 5sd, and 98.7% then were able to spike.

As seen from the corner plot, we did not attempt to adjust the bounds of the parameter space precisely in this example, but more work could be done to refine this interneuron model to perform comparisons with other electrical types.

#### Electrical features

In Table S1 we list the electrical features evaluated with eFEL library (https://github.com/BlueBrain/eFEL) on experimental and numerical traces to compute the scores and model cost. We refer the interested reader to^42^ for more details on the protocols and features.

## Data Availability

• Data have been deposited at Zenodo and Harvard Dataverse and are publicly available as of the date of publication. Accession numbers are listed in the key resources table. • All original code has been deposited at Zenodo and is publicly available as of the date of publication. DOIs are listed in the key resources table. The code is also available on Github at https://github.com/BlueBrain/emodel-generalisation or on pypi.org at https://pypi.org/project/emodel-generalisation/0.1.1/. • Any additional information required to reanalyze the data reported in this paper is available from the lead contact upon request.
