# Supplementary material for: Controlling morpho-electrophysiological variability of neurons with detailed biophysical models
Source: iScience. 2023 Oct 16;26(11):108222. doi: 10.1016/j.isci.2023.108222 (PMC10638024; doi:10.1016/j.isci.2023.108222)
Supplement: Document S1. Figures S1–S11 and Table S1 [file mmc1.pdf]

iScience, Volume *26*

Supplemental information

Controlling morpho-electrophysiological variability of neurons with detailed biophysical models

Alexis Arnaudon, Maria Reva, Mickael Zbili, Henry Markram, Werner Van Geit, and Lida Kanari

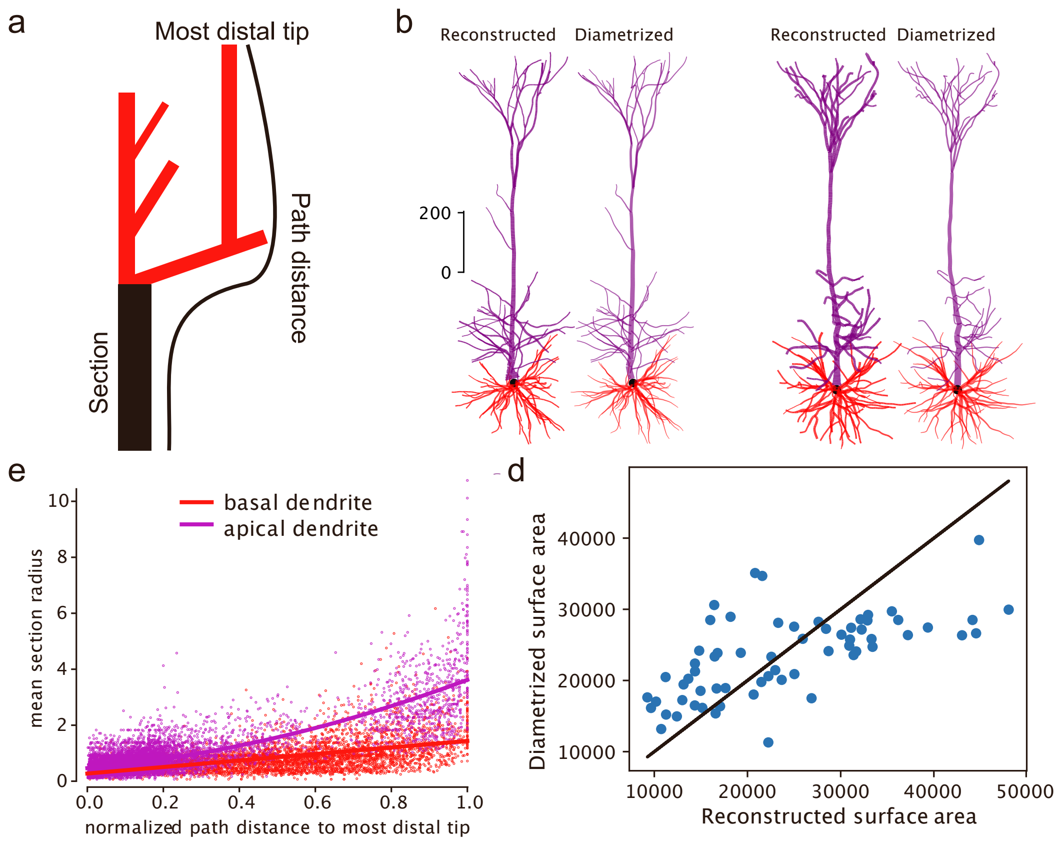

Figure S1: **Diametrization algorithm, Related to Fig. 1. a.** Illustration of distance computation used in the diametrization model for the section in black. **b.** Example of two reconstructed morphology (left) and its rediametrized versions (right) **c.** Mean section diameters as a function of path distance to the most distal terminal section for the population of thick-tufted morphologies, with a fit per type of neurite. **d.** Comparison of surface areas of all dendrites form 0 to 500μm between original and diametrized morphologies.

**E-type**	**Protocol**	**E-features**
cADpyr L5	APWaveform 320 %	AP_amplitude, AP1_amp, AP2_amp, AP_duration_half_width, AHP_depth
	IV -100 %	voltage_deflection, voltage_deflection_begin
	IDrest & IDthresh 150, 200, 280 %	voltage_base, voltage_after_stim, AP_amplitude, APlast_amp, AHP_depth, inv_time_to_first_spike, time_to_last_spike, inv_first_ISI, inv_second_ISI, inv_third_ISI, inv_fourth_ISI, inv_fifth_ISI, mean_frequency
	SpikeRec_600 %	decay_time_constant_after_stim, voltage_after_stim, Spikecount
	IV -20 % (Rin)	ohmic_input_resistance_vb_ssse, voltage_base
	IV 0 % (RMP)	voltage_base, Spikecount
	RinHoldCurrent	bpo_holding_current
	Threshold	bpo_threshold_current
	bAP	Spikecount, maximum_voltage_from_voltagebase, maximum_ca_prox_apic_from_voltagebase, maximum_ca_prox_basal_from_voltagebase, maximum_ca_prox_soma_from_voltagebase, maximum_ca_prox_ais_from_voltagebase
cNAC	IDThresh/IDrest 150, 200, 250, 300 %	voltage_base, voltage_after_stim, AP_amplitude, APlast_amp, AHP_depth, inv_time_to_first_spike,

		time_to_last_spike, inv_first_ISI, inv_second_ISI, inv_third_ISI, inv_fourth_ISI, inv_fifth_ISI, mean_frequency
	APWaveform 360 %	AP_amplitude, AP1_amp, AP_duration_half_width, AHP_depth
	IV -100 %	voltage_deflection, voltage_deflection_begin
	IV -20 % (Rin)	ohmic_input_resistance_vb_ssse, voltage_base
	IV 0 % (RMP)	voltage_base, Spikecount
	RinHoldCurrent	bpo_holding_current
	Threshold	bpo_threshold_current

Table S1: List of protocol and features, Related to Fig. 2.

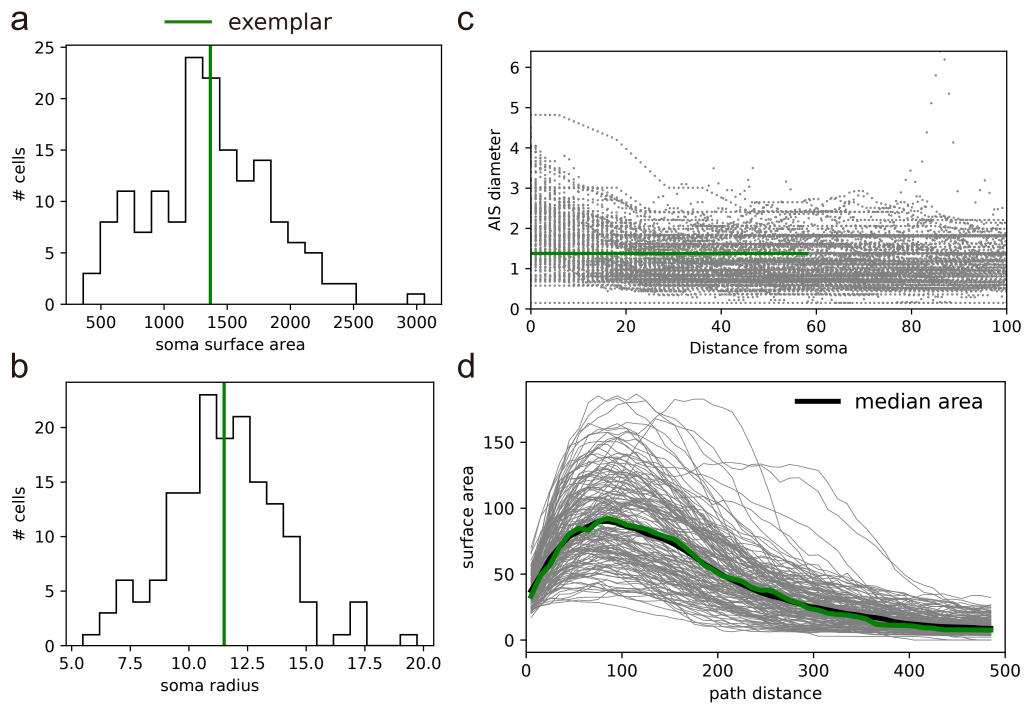

Figure S2: **Construction of exemplar morphology, Related to Fig. 1. a.** Distribution of soma surface areas of all L5 pyramidal cells, with the chosen area in green. **b.** Distribution of soma radii of all cells with chosen radius in green. **c.** Diameters of the reconstructed points of the first sections of the axon, interpreted as AIS diameters. In green is the average AIS diameter of the first $60\mu m$ used for the exemplar. **d.** Proximal surface area profile of all dendrites, computed with 50 bins in path distance. The median profile is in black, and the closest to it is the choice of exemplar dendrites (in green).

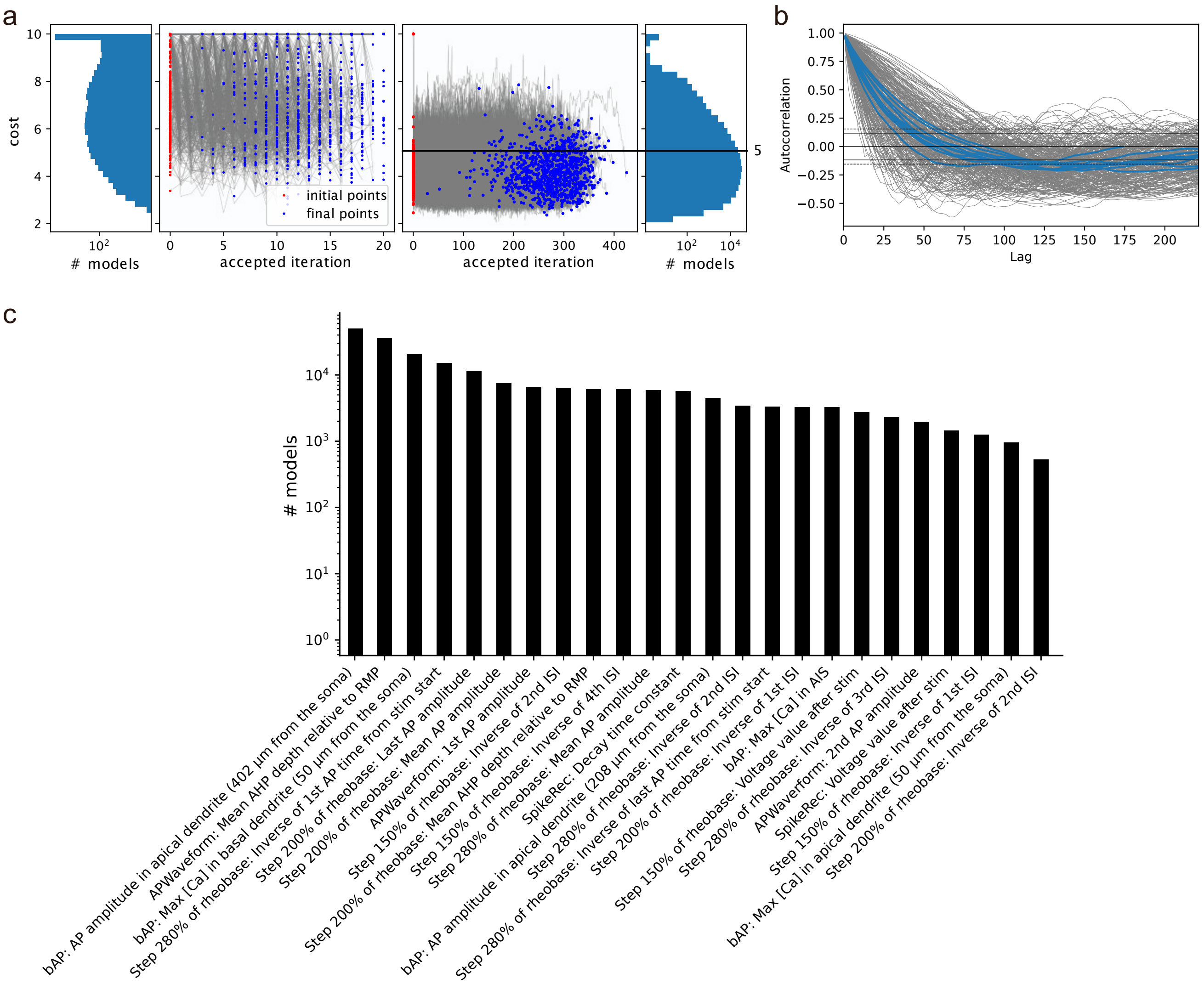

Figure S3: **MCMC additional figure, Related to Fig. 2. a.** Cost convergence for burn-in and main MCMC run. Red dots correspond to the initial costs of each chain, grey lines are the chain costs trajectories, and blue dots correspond to the final cost when the chain was stopped after 500 iterations. The x-axis corresponds to the accepted iterations, thus the horizontal scatter of blue points represents the variability in acceptance rates and an average acceptance rate of around 60%. The left and right panels are respectively the distributions of costs for the burn-in phase (left middle panel) and main MCMC run (right middle panel). **b.** Auto-correlation of all the chains in grey, and 10 chains in blue. Horizontal lines show significance intervals, as defined in Panda's auto-correlation plot function. **c.** Distributions of scores saturating the cost for all models with costs < 5 (marked with a horizontal black line in panel **a**).

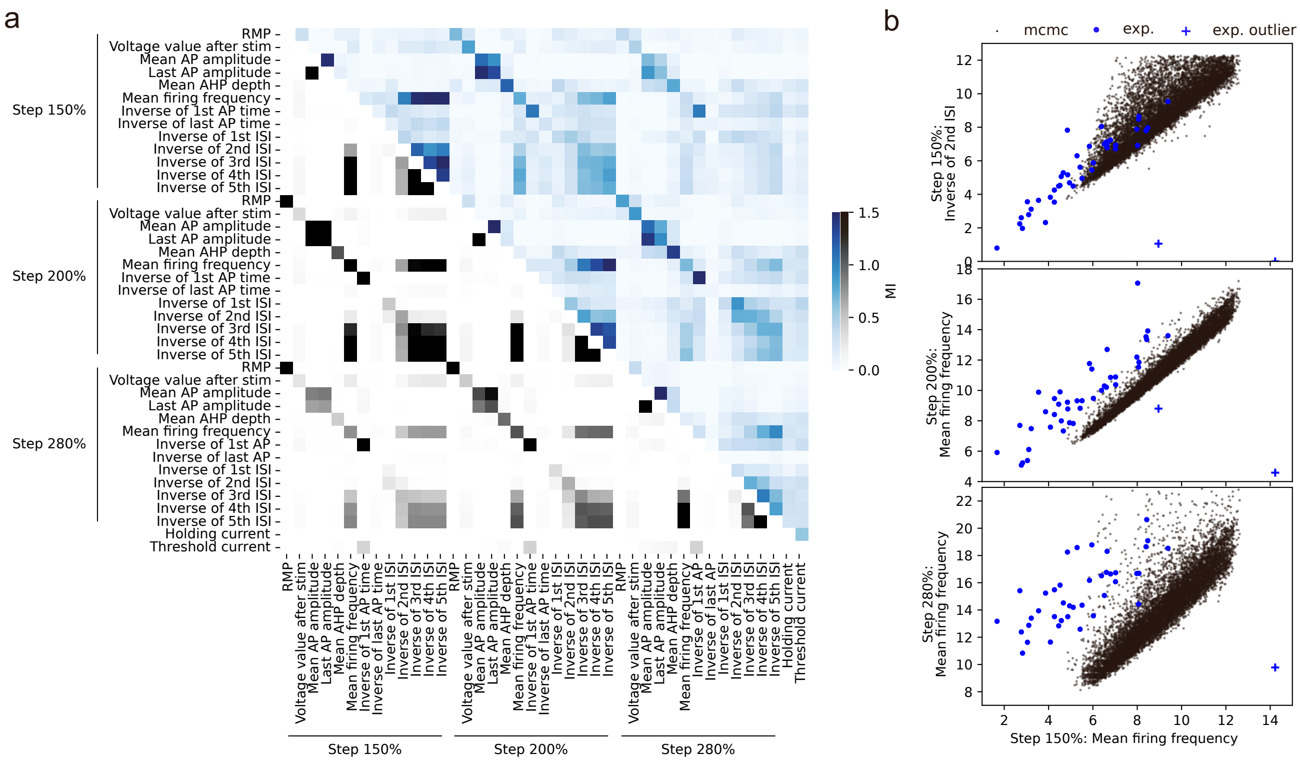

Figure S4: **Correlations of experimental and MCMC features, Related to Fig. 2. a.** Correlations (Mutual information) between features in experimental data (blue) and MCMC samples (black) for all samples with costs < 3. **b.** Scatter plots between some pairs of features, showing differences and similarities between experimental data and the MCMC sampling. Outliers have been discarded to make a correlation matrix.

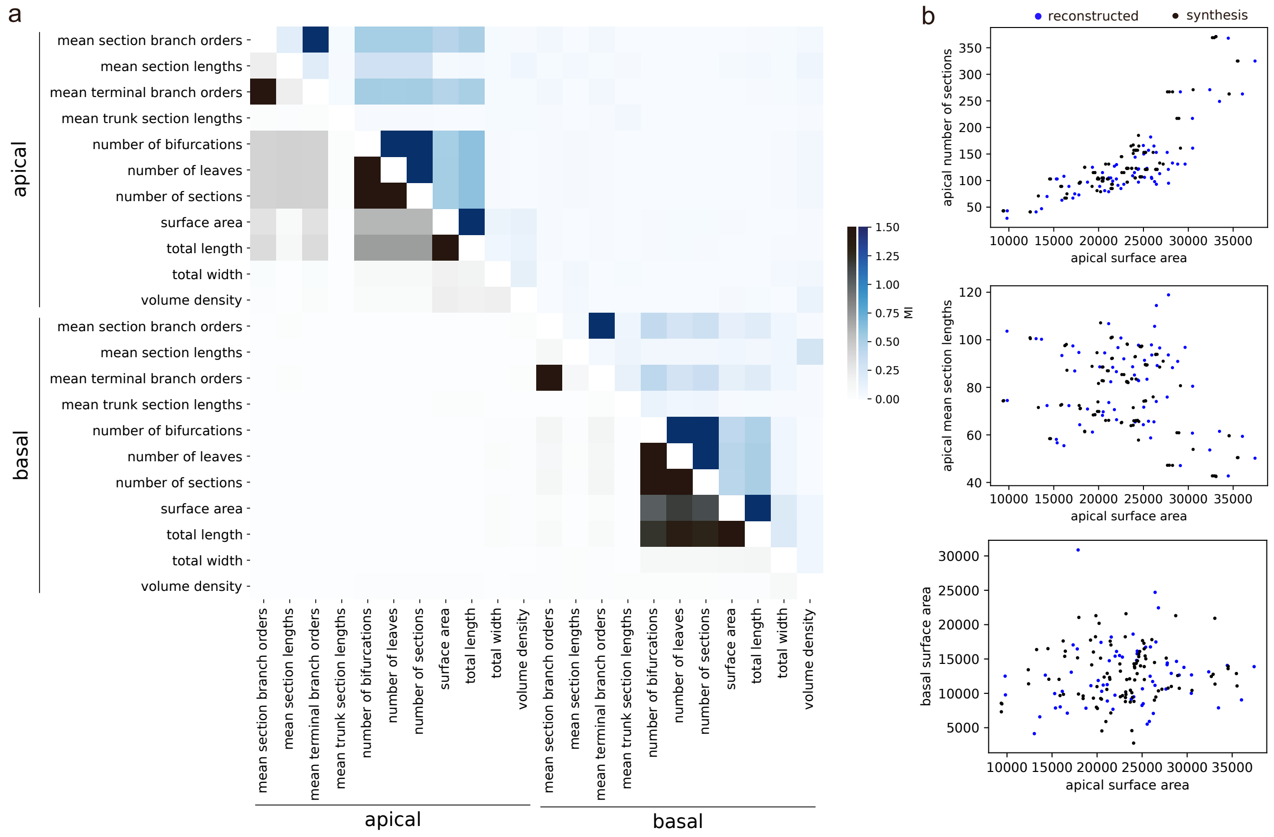

Figure S5: **Correlations of morphological features. Related to Fig. 1. a.** Correlations (Mutual information) between some morphological features in reconstructed (blue) and synthesised (black) morphologies. **b.** Scatter plots between some pairs of features.

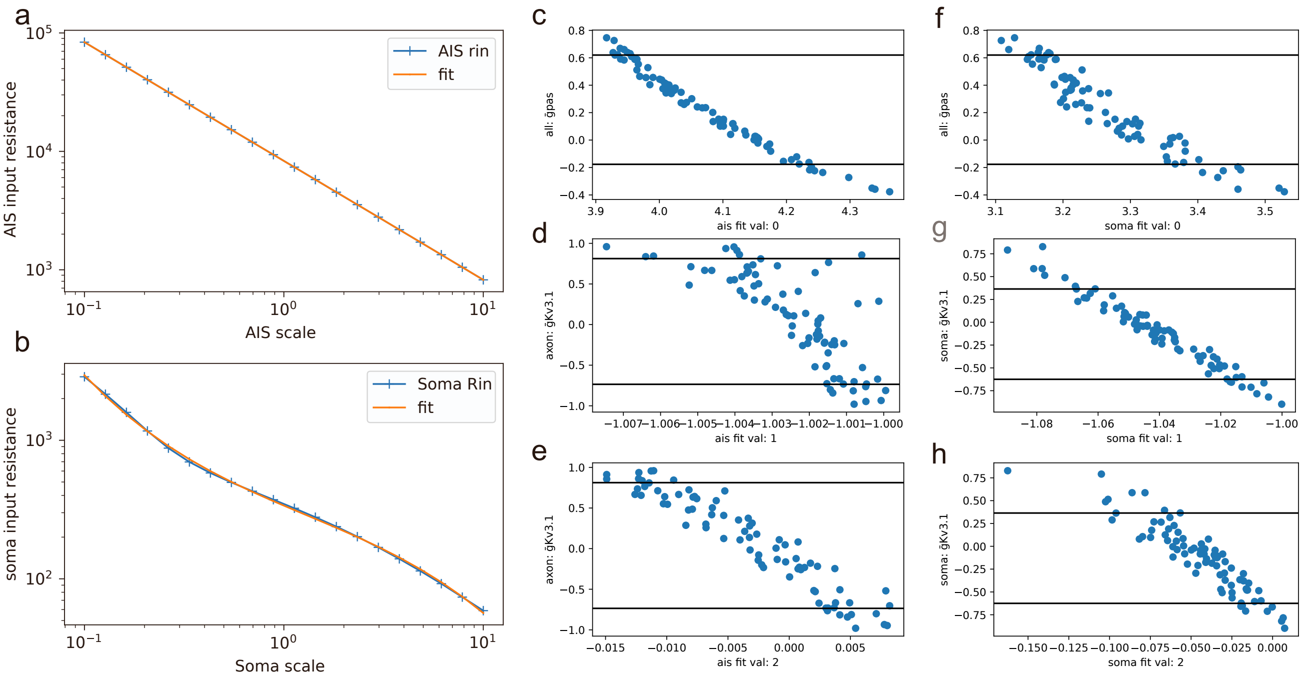

Figure S6: **Soma and AIS input resistance models, Related to Fig. 3. a** AIS input resistances for model (blue) used in Fig. **??** and a cubic polynomial fit (orange). **b** Somatic input resistances for model (blue) used in Fig. **??** and a cubic polynomial fit (orange). **c-e** Mostly correlated model (normalized from -1 to 1) parameters with the first three fit parameters for the AIS resistance model. Black lines are 10 and 90 percentile on the parameter values. **f-h** Mostly correlated model parameters with the first three fit parameters for the soma resistance model. Black lines are 10 and 90 percentile on the parameter values.

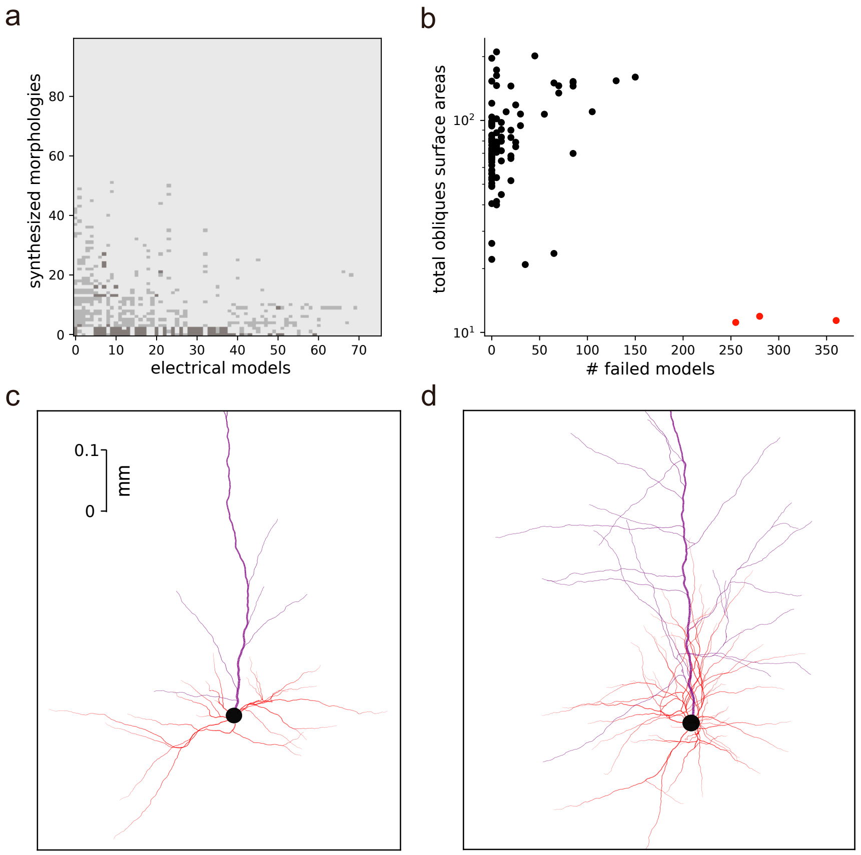

Figure S7: **Generalisation with synthesised morphologies, Related to Fig. 3.** **a** Selection matrix with selected models and synthesised morphologies generated from selected morphologies. Grey pixels correspond to scores above 5 and black for scores above 10. White correspond to scores below 5. **b** For each morphology, we compute the total surface areas of obliques, which is a good predictor of failed morphologies. **c** We show a zoom on the oblique region of a failed morphology. **d** Similar zoom as in **c** but for a working morphology.

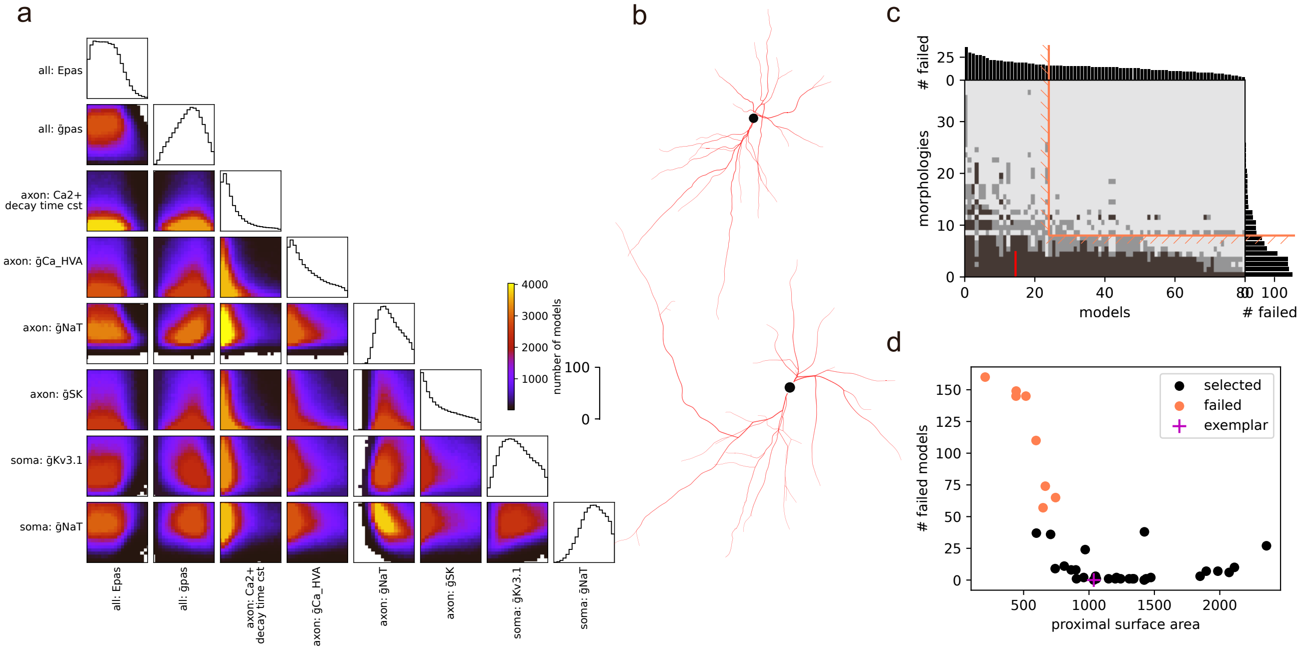

Figure S8: **cNAC electrical model, Related to Fig. 2.** **a** Corner plot of most correlated parameters of an MCMC run on cNAC electrical model based on (**?**). **b** Exemplar morphologies used for MCMC (left) with a layer 2/3 Martinotti cell and generalisation (right) on layer 5 Martinotti cell. **c** Selection matrix for layer 5 Martinotti cells. **d** Proximal surface areas of cells that are selected and not selected.

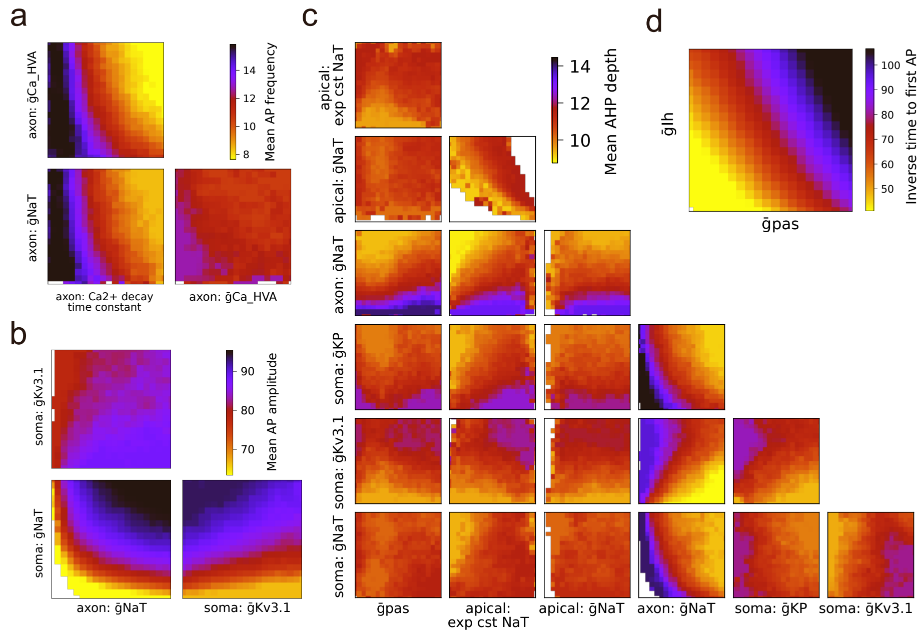

Figure S9: **Corner plot with average features values, Related to Fig. 2.** We plot the average feature values for additional features from Fig. **??**f-g. Tee features are: mean AP frequency in **a**, mean AP amplitude in **b**, mean AHP depth in **c** and inverse time to first AP in **d**.

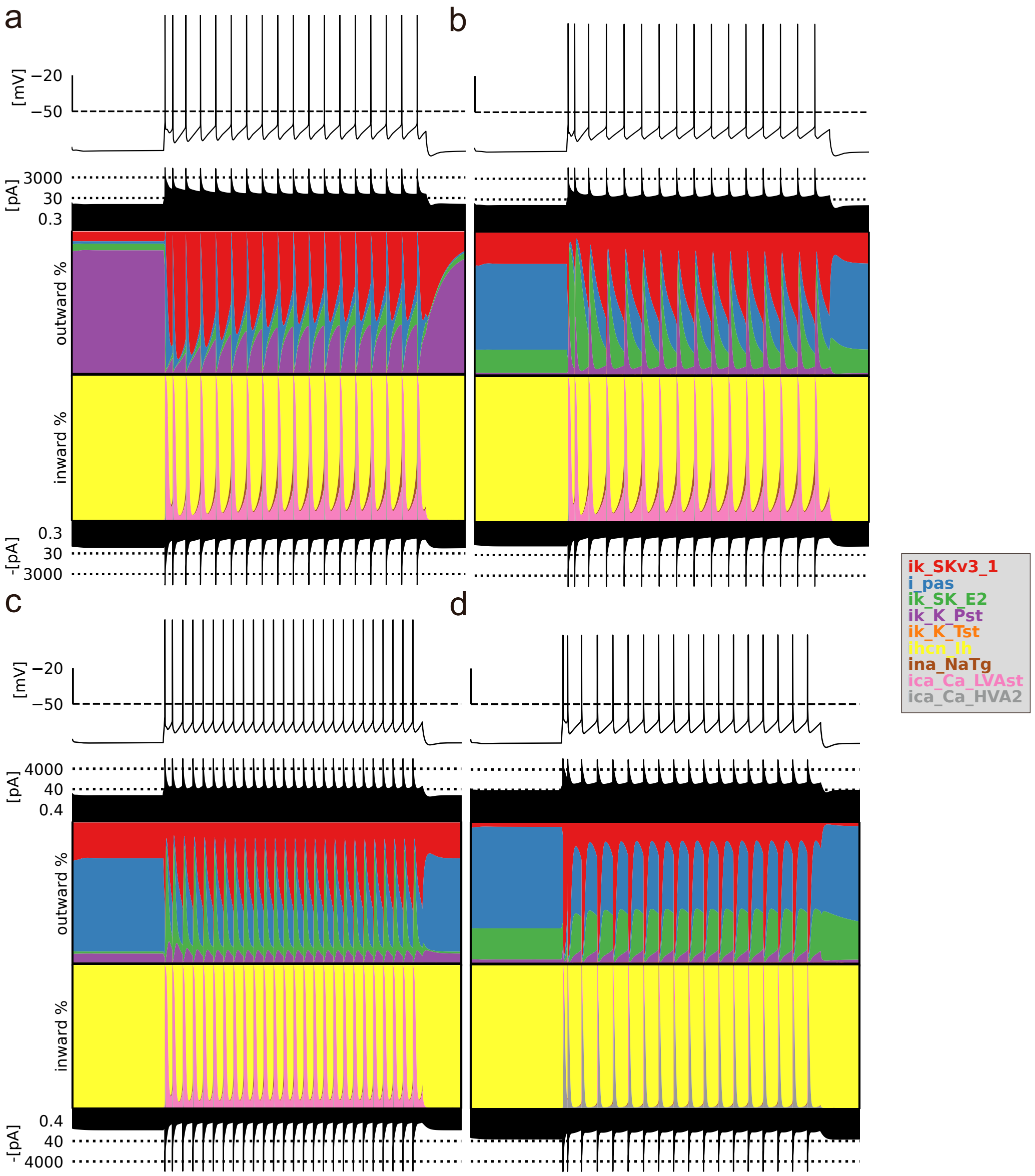

Figure S10: **Currentscape of four MCMC models, Related to Fig. 2.**. Currentscape are made following (**?**) and represent the various inwards and outwards currents present in the cell during activity with the following models from Fig. **??**: blue in **a**, red in **b**, magenta in **c** and green in **d**.

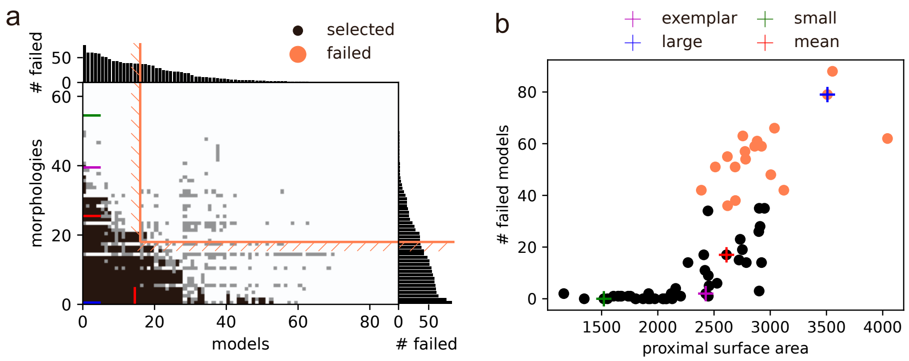

Figure S11: **Selection of model and morphologies without AIS/soma adaptation, Related to Fig. 3.** Same panels as Fig. **??**a-b, but without adaptation of AIS/soma.
